# Growth hormone treatment in congenital tufting enteropathy: a case report and literature review

**DOI:** 10.3389/fendo.2024.1492297

**Published:** 2025-01-30

**Authors:** Mehmet Ali Oktay, Mahmut Orhun Çamurdan, Ödül Eğritaş Gürkan, Başak Alan Tehçi, Esra Döğer, Aysun Bideci

**Affiliations:** ^1^ Department of Pediatric Endocrinology, Faculty of Medicine, Gazi University, Ankara, Türkiye; ^2^ Department of Pediatric Gastroenterology, Faculty of Medicine, Gazi University, Ankara, Türkiye

**Keywords:** congenital tufting enteropathy, growth hormone therapy, parenteral nutrition, short stature, growth

## Abstract

This article aims to evaluate the effects of growth hormone (GH) therapy in a case with congenital tufting enteropathy (CTE). CTE is a rare autosomal recessive enteropathy that typically presents with persistent diarrhea. In this case, a 13-year-old girl presented with a diagnosis of CTE. Due to short stature, GH therapy was considered. Pre- and post-treatment evaluations were conducted for height, growth rate, and motor skills. As a result, an increase in growth rate and improvement in motor skills were observed with GH therapy. These findings suggest that the potential of GH therapy is to increase growth and improve the quality of life in patients with CTE. Further studies are needed to evaluate the long-term effects of GH therapy and its efficacy in broader patient groups.

## Introduction

Congenital tufting enteropathy (CTE) is a rare autosomal recessive enteropathy that typically presents early in life and is often characterized by persistent diarrhea. In some severe cases, total parenteral nutrition (TPN) may be required, and in extreme situations, small bowel transplantation may be necessary. Mutations in the human epithelial cell adhesion molecule (EpCAM) gene have been reported in the typical form of CTE, characterized by isolated refractory diarrhea (1, 2). In CTE cases, standard deviation scores (SDS) for height and body mass index (BMI) are typically low. Following the initiation of TPN, an increase in insulin-like growth factor 1 (IGF-1) levels occurs, leading to an increase in height SDS (3). However, to date, there have been no reported cases evaluating the effects of growth hormone (GH) therapy in CTE patients without parenteral nutrition. This article presents the experience with GH therapy in a case of CTE.

## Case presentation

A 13-year-old female patient, born prematurely at 34 weeks with a birth weight of 1800 grams, was referred to our clinic for short stature associated with tufting enteropathy. Genetic testing revealed a homozygous c.556-14A>G mutation in the EpCAM gene. The patient developed persistent diarrhea in the first two weeks of life and required TPN for six years, resulting in prolonged hospitalization. She was on TPN for six years but is no longer receiving TPN. Currently, she is only receiving oral feedings, which include specialized nutritional supplements to meet her dietary needs. The patient’s condition was initially managed with steroid treatment, which effectively controlled the symptoms of CTE, but it was not required for the two years prior to the evaluation.

On physical examination, her body weight was 21.5 kg (-6.01 SDS), height was 106 cm (-8.81 SDS), and BMI was 19.1 (-0.87 SDS). She had an atypical facial appearance, was prepubertal (Tanner stage 1), and could walk with support but had weak muscle strength, could not speak, and could not feed herself. Her parents’ heights were 176 cm and 156 cm, with a mid-parental height (MPH) of 159.5 cm.

Growth hormone stimulation tests revealed peak GH values of 1.91 ng/ml and 2.95 ng/ml with levodopa and clonidine, respectively. The level of insulin-like growth factor-1 (IGF-1) was measured at 163.8 ng/ml (reference range: 146-480 ng/ml). IGF-1 levels were not extremely low. Other laboratory parameters were normal. The GH stimulation test was performed unprimed. Other laboratory parameters were within normal limits, and thyroid function tests and celiac screening were normal. Karyotyping was performed to rule out Turner syndrome, which was negative. Gonadotropin levels were as follows: LH <0.1 mIU/mL, FSH 0.2 mIU/mL, consistent with Tanner stage 1. Cranial magnetic resonance imaging showed no abnormalities. Her bone age was assessed as six years according to the Greulich and Pyle atlas (4), despite her chronological age of 13 years and three months.

Growth hormone therapy was initiated at a dose of 0.033 mg/kg/day and continued for one year. Based on growth rate and IGF-1 level evaluations, GH was administered at an average dose of 0.037 mg/kg/day. During the treatment period, the patient’s body weight remained stable. This was likely due to suboptimal oral intake, which did not fully meet her nutritional needs despite specialized nutritional supplements. The treatment led to significant growth, with the patient reaching a height of 115.8 cm (-7.8 SDS) at 14 years and nine months of age. Her growth velocity prior to GH therapy was 2.1 cm/year, compared to 8.5 cm/year during the first year of GH therapy, according to our measurements (**Table 1**). The corrected Z-score for bone mineral density was -4.69, although bone metabolism parameters were normal. Vitamin D and calcium supplementation were continued throughout treatment. No serious adverse effects were observed during GH therapy.

**Table 1:** Before and after Growth Hormone (GH) therapy: anthropometric measurements.

	Chronological Age	Weight (kg) (SD)	Height (cm) (SD)	BMI (kg/m2) (SD)
Initial Presentation	13 years 3 months	21,5 (-6,01)	106 (-8.81)*	19,1 (-0,87 )
6 months after initial presentation (GH initiation)	13 years 10 months	19,1 (-6,23)	107,3 (-8,38)*	16,5 (-1,45)
3.5 months after GH initiation	14 years 2 months	20,5 (-7,79)	108,7 (-8,79) 115 (-7,76)*	17,3 (-1,58)
7 months after GH initiation	14 years 5 months	21 (-7,88)	113,2 (-8,12)	16,3 (-2,26)
9 months after GH initiation	14 years 7 months	21,5 (-7,93)	113,5 (-8,10)	16,6 (-2,16)
11 months after GH initiation	14 years 9 months	21,5 (-8,13)	115,8 (-7,81)	16,0 (-2,68)

GH, Growth Hormone, * Height value measured lying down, SD, Standart Deviation; BMI, Body Mass Index.

After one year of GH therapy, the patient demonstrated significant improvements. Her height increased by 8.5 cm/year, and she gained 1 SDS in height. Additionally, her motor skills improved; she could stand unsupported and take steps independently. Her bone age advanced to six years and six months, with normal bone metabolism parameters.

The patient continues to receive regular follow-up to monitor growth, development, and any potential side effects of GH therapy. IGF-1 levels after one year of GH therapy were 297 ng/ml (146-480 ng/ml). No serious adverse events have been reported during the follow-up period.

## Discussion

Intestinal failure (IF) refers to a reduction in the absorption of fluids and nutrients due to dysfunction of the gastrointestinal tract. In severe cases of IF, TPN is used as a fundamental supportive treatment (5). Causes of growth failure in IF include chronic inflammation, malnutrition, genetic potential, and glucocorticoid treatments (6). In children with chronic refractory IF requiring long-term TPN, abnormal physical development such as decreased linear growth, low weight gain, bone abnormalities, and delayed puberty can be observed (6).

A study of CTE cases showed that IGF-1 levels were low at diagnosis and increased in most cases after the initiation of TPN. However, it was reported that the growth rate did not reach a sufficient level in these cases (3). Similarly, in our case, it was observed that the growth rate remained low even after TPN therapy, and it was far below the targeted SDS value.

**Figure 1:**
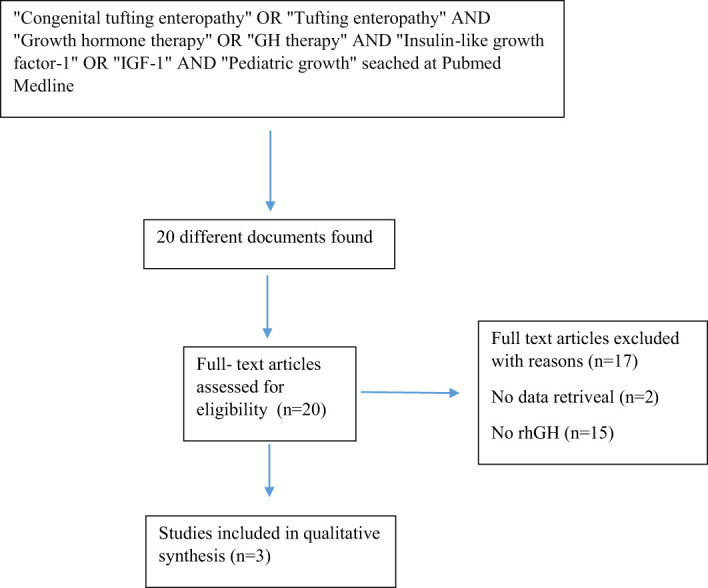
The appearance of the patient after 1 year of GH treatment.

There are very few articles in the literature on the effects of growth hormone therapy in CTE cases (**Figure 1**). Zhou et al. reported weight gain in a male infant with CTE after short-term GH therapy, but the long-term effects were unknown (2). In our case, short-term GH therapy was administered without receiving TPN, and the therapeutic impact on growth rate and height was observed.

In a reported case of CTE by El-Matary et al., it was stated that growth stopped in the second year, and significant abnormalities were observed in skeletal radiographs (7). However, bone metabolism parameters were normal in our case, and no signs of skeletal dysplasia were observed.

There are several reports indicating that GH treatment is effective in the short term for growth disorders caused by various intestinal diseases in children. Our findings are consistent with these reports, highlighting the potential of GH therapy to improve growth outcomes in CTE.

When GH or IGF-I is administered alone in parenteral nutrition models, similar increases in serum IGF-I concentration are observed. GH has been reported to increase skeletal muscle mass, while IGF-I attenuates intestinal atrophy and abnormal intestinal ion transport caused by TPN (3, 8).

It has been shown that small for gestational age (SGA) patients who achieve catch-up growth have better motor skills compared to those who do not (9). There is some evidence that intellectual performance improves during GH treatment in SGA patients (10, 11). In this context, effects similar to those observed in SGA patients  were also observed in terms of physical and intellectual aspects in our case.

Our patient responded well to GH therapy with an increased growth rate and improved motor skills. GH therapy may be effective in improving the growth rate and quality of life in asymptomatic CTE patients. To date, only short-term effects of GH therapy have been reported in CTE patients in the literature. However, further studies involving larger patient groups are needed to evaluate the impact of this treatment.

### Learning points

- Growth hormone therapy: Can lead to significant improvements in growth rates and motor skills in CTE patients, as demonstrated in this case.
- Limited literature: Few reports exist on GH therapy in CTE, emphasizing the need for further studies to assess long-term effects and outcomes.
- Quality of life: GH therapy may enhance physical and intellectual development, improving the overall quality of life for CTE patients.

## Research strategy

A specialized search string was performed in PubMed Medline using separate keywords. Keywords were combined in different combinations using the “AND” database with appropriate filters to maximize the identification of articles explaining the connection between growth and CTE. Specifically, a PubMed search was conducted with the search string: “Growth” AND “tufting enteropathy.” The search included all articles published until February 2024.
